# The Effect of Silage Microbial Inoculants on the Silage Quality of WL358HQ Alfalfa

**DOI:** 10.3390/microorganisms13051026

**Published:** 2025-04-29

**Authors:** Siyi Wang, Zhennan He, Yuanyuan Jing, Le Sun, Guolin Yang, Bin Liu, Fengqin Gao

**Affiliations:** 1 Institute of Grassland Research, Chinese Academy of Agricultural Science, Hohhot 010010, China; 17860788357@163.com (S.W.); a2964898754@163.com (Z.H.); jingyuanyuan@caas.cn (Y.J.); 16634230300@163.com (G.Y.); 2 College of Grassland Science, Qingdao Agricultural University, Qingdao 266109, China; 15066745843@163.com; 3 Animal Husbandry Institute, Inner Mongolia Academy of Agricultural & Animal Husbandry Sciences, Hohhot 010010, China

**Keywords:** WL358HQ Alfalfa, silage, fermentation quality, nutritional quality

## Abstract

In order to explore the effect of different silage microbial inoculants on the silage quality of WL358HQ Alfalfa (*Medicago sativa* L.), the test consists of the following six treatments: Xinlaiwang I—straw silage inoculant (A), Xinlaiwang I—alfalfa silage inoculant (B), Zhuang Le Mei silage inoculant (C), Baoshiqing (D), KOFASIL S lactic acid bacteria silage inoculant (E) and distilled water (F). Nutritional and fermentation indexes were determined after 60 days of natural fermentation at room temperature. The results showed that the contents of crude protein (CP) and water-soluble carbohydrate (WSC) of the B treatment were higher than other treatments. The Relative Feed Value (RFV) of the B treatment was high, the lactic acid (LA) content of the B treatment was significantly increased (*p* < 0.05) and the contents of neutral detergent fiber (NDF), acid detergent fiber (ADF), pH and ammoniacal nitrogen/total nitrogen (AN/TN) of the B treatment were low. The results showed that treatment B has the best ensilage effect and the highest ensilage membership function, which can effectively improve the quality of WL358 alfalfa silage.

## 1. Introduction

Alfalfa is a perennial high-quality leguminous forage [1]. It has a well-developed root system, which can fully absorb soil nutrients [2], weaken surface runoff and protect water and soil [3]. Its biological nitrogen fixation ability is strong and it has wide adaptability [4]. It is characterized by cold resistance, drought resistance and salt-alkali tolerance. With its strong stress resistance, regeneration ability and extensive adaptability, it has received wide attention and promotion worldwide. Alfalfa has a cultivation history of more than two thousand years in China and is an essential feed resource. The utilization of processed alfalfa in feed can prominently enhance the production performance of animals [5] and the quality of animal products.

At present, the production and processing methods of alfalfa include green forage, the production of green hay, grass powder and silage, as well as the production of compound feed that is preferred by both livestock and poultry [6]. The predominant modulation and processing approach of alfalfa is the production of hay. Nevertheless, during the modulation and storage of hay, external environmental factors such as rain [7], fallen leaves [8] and microorganisms are highly likely to result in a substantial loss of nutrients in the hay. Furthermore, unfermented alfalfa contains a considerable amount of nutrients and crude fiber. The elevated content of crude fiber makes it arduous for nutrients to be absorbed [9] and simultaneously confronts the challenge of difficult short-term storage. Conversely, alfalfa silage can mitigate the loss of nutrients and the volume occupied during storage and transportation. Hence, in order to prolong the feed supply cycle for ruminants and guarantee the availability of nutrient-rich feed even in non-growing seasons, it is indispensable to explore efficient alfalfa silage techniques.

Due to its high buffering capacity, alfalfa, a leguminous plant [10], frequently encounters issues with unstable fermentation quality when used as a single raw material for silage. To overcome this technical bottleneck, additional measures such as adding acidifiers, promoting lactic acid fermentation or mixing with other plants with low buffering capacity value are usually needed during storage and fermentation to solve this problem. Thus, the development of efficient silage fermentation technologies has emerged as a crucial area of research [11]. He Lichao et al. [12] demonstrated that *Lactobacillus plantarum* is the most suitable for the preparation of mixed silage of alfalfa and rapeseed straw. *Lactobacillus plantarum* can significantly improve the silage fermentation quality (*p* < 0.05). Yan et al. [13] demonstrated that alfalfa raw materials with a moisture content of 55% and supplemented with 0.4% propionic acid (PA) have better overall ensiling performance and can significantly improve the quality of silage (*p* < 0.05). Xinlaiwang I—alfalfa silage inoculant is a highly efficient biological agent. The abundant lactic acid bacteria in it can significantly increase the content of lactic acid, reduce feed pH, reduce feed spoilage and nutrient loss [14], reduce the content of cellulose and anti-nutrient factors in feed, improve the storage stability of silage and improve the utilization rate of protein and heat energy of silage by livestock. Xinlaiwang I—straw silage inoculant is an efficient silage additive containing *Lactobacillus plantarum*, *Pediococcus pentosus* and *Lactobacillus brucei*, which can rapidly ferment to produce lactic acid, reduce pH value, inhibit the growth of mold and spoilage bacteria, thus reducing the loss of nutrients and dry matter, and improve the quality of silage. The Zhuang Le Mei silage inoculant is also an efficient silage additive, which contains *Lactobacillus plantarum* and *Lactobacillus brucei*, which can quickly produce lactic acid and rapidly reduce pH, thus inhibiting the activity of spoilage bacteria, reducing the loss of nutrients and making feed nutrients better preserved. Baoshiqing contains highly efficient homotype and heterotype fermented lactic acid strains such as *Lactobacillus plantarum*, *Pediococcus pentosus* and *Lactobacillus brucei*. Some scholars [15] found that the dry matter content and lactic acid content of the treatment with *Lactobacillus plantarum*, *Lactobacillus brucei*, *Pediococcus pentosus* and *Lactobacillus kimchi* were significantly higher than those without additives. KOFASIL S lactic acid bacteria silage inoculant (E) is an environmentally friendly and effective silage additive, containing carefully selected non-transgenic lactobacillus strains, which can produce lactic acid and acetic acid, inhibit the growth of yeast and mold, improve the aerobic stability of silage, so that the silage will not be heated twice even after exposure to air, and maintain anti-fungal properties. Different additives have different effects on the microbial community structure in the fermentation process. Through research, the most efficient additives that promote the growth of beneficial bacteria and inhibit the development of harmful bacteria can be screened out, optimizing the fermentation process and providing a scientific basis for silage production. Shi et al. showed [16] that mixing 10% forsythia with alfalfa in silage can significantly improve silage quality, increase aerobic stability and reduce mycotoxin content. Adding 5 mg/kg *Lactobacillus plantarum* (LP) and 75 mg/kg cellulase (CE) to alfalfa silage had the best effect on fermentation quality and nutrient composition [17]. A 10% grape pulp (GRP) supplementation significantly increased crude protein (CP) [18], yeast colony count and butyric acid levels in alfalfa silage. On the contrary, adding 10% orange pulp increased the levels of water-soluble carbohydrate (WSC) and valeric acid and decreased the levels of acetic acid in alfalfa silage samples. The main phospholipid content and polyunsaturated fatty acid composition in raw milk can be increased by feeding alfalfa silage diet to cows, which is more beneficial to human health [19]. Cookies past their sell-by date can be used as silage additives in alfalfa silage. It was determined that it was appropriate to add 4% expired cookie waste to alfalfa silage [20]. Studies have shown [21] that silage with good quality can be prepared by adjusting the moisture content of raw materials and using silage additives such as *Lactobacillus*. The commonly used *Lactobacillus* includes *Lactobacillus*, *Pediococcus*, *Lactococcus*, *Enterococcus*, *Streptococcus*, *Leuconostoc* and *Bifidobacterium*.

Five commercially available silage agents (Xinlaiwang I—straw silage (A), Xinlaiwang I—alfalfa silage (B), Zhuang Le Mei silage starter culture (C), Baoshiqing (D), Kangfuqing S lactic acid bacteria silage (E)) were systematically compared for the first time, covering a wider range of lactic acid bacteria genera. It has enriched the categories of *Lactobacillus*, *Pediococcus*, *Lactococcus* and *Enterococcus* commonly used in silage research. The nutritional and fermentation performance data of different silage agents in specific crops (WL358HQ Alfalfa) were provided through the actual silage effect evaluation, which provided the scientific basis for the optimal selection and customized application of silage, aiming to explore the effects of various inoculants on the fermentation performance of silage and the selection of the most suitable inoculant for alfalfa silage. This study posits the hypothesis that “Xinlaiwang I—alfalfa silage inoculant” is the optimal choice.

## 2. Material and Methods

With the WL358HQ Alfalfa variety as the target, the experiment was carried out at the Knights Dairy alfalfa planting base in Dalat Banner, Ordos City, under specific production location and climatic conditions, Inner Mongolia on 10 July 2020. The region is located in a typical temperate continental monsoon climate zone, with an average annual temperature of 6.8 °C, an extreme minimum temperature of −30.9 °C, a frost-free period of 135 days and an average annual precipitation of 320 mm, of which the growing season (April–September) precipitation accounts for 89% of the year. The altitude is 1010 m, the soil is chestnut soil, the content of organic matter is 4.86 g/kg, the content of available potassium is 94.65 mg/kg, the content of available phosphorus is 10.46 mg/kg, the content of alkaline hydrolytic nitrogen is 11.15 mg/kg and the pH value is 8.2. Sprinkler irrigation conditions are used to eliminate the effects of drought and keep the soil low in salts and other pollutants. The drying process and F: Harvesting take place at the beginning of alfalfa flowering and the stubble height is usually between 7 and 10 cm. The drying process is carried out naturally; the cut plants are spread out in a well-ventilated, sunny area and turned regularly to accelerate water evaporation and ensure uniform drying. The drying time depends on the weather conditions and the moisture content of the plant until the moisture content drops to about 60%. Table 1 shows the nutrient composition of WL358HQ Alfalfa.

### 2.1. Experimental Design

The experiment was designed with six treatments. Among them, five treatments were microbial inoculant treatments, specifically, the addition of Xinlaiwang I—straw silage inoculant, Xinlaiwang I—alfalfa silage inoculant, Zhuang Le Mei silage inoculant, Baoshiqing, and KOFASIL S lactic acid bacteria silage inoculant, along with an F treatment (F) treated with distilled water. The main components and sources of each microbial inoculant are presented in Table 2. After the alfalfa was mowed, it was aired until the moisture content reached approximately 60% and then chopped into 1–2 cm pieces. The chopped alfalfa raw materials were thoroughly mixed with the microbial inoculants. In accordance with the experimental design, the compound lactic acid bacteria microbial inoculant was sprayed onto the surface of the raw materials (the F treatment was sprayed with an equivalent amount of distilled water), thoroughly mixed and kneaded to ensure uniform spraying and placed in vacuum silage bags (200 mm × 250 mm). Each bag weighed approximately 300 g, with three replicates for each treatment. Dosage of inoculants is shown in Table 2. Using a vacuum machine for packaging, a total of 18 samples were sealed. Under normal temperature conditions, after 60 days of fermentation, the bags were opened to analyze their fermentation quality and nutritional components. No water needs to be added. Air dry and then add bacteria directly. To ensure the reliability and robustness of our results, the experiment was conducted with three replicates for each experimental group, enhancing statistical significance and reproducibility.

Xinlaiwang I straw silage inoculant: specifically designed for straw silage, it effectively enhances the fermentation quality of straw and increases its nutritional value. Xinlaiwang I alfalfa silage: optimizing the fermentation process of alfalfa for alfalfa silage to maintain its nutrition and taste. Zhuanglemei silage inoculant: a multifunctional silage inoculant suitable for various feed crops, enhancing the stability and palatability of silage. Baoshiqing: emphasize rapid fermentation and long-term preservation to reduce nutrient loss during the silage process. KOFASIL S lactic acid bacteria silage inoculation liquid: it contains specific lactic acid bacteria strains, promotes high-quality lactic acid fermentation and ensures the safety and nutrition of silage feed.

### 2.2. Determination Indexes and Methods

After 60 days of airtight fermentation, the moldy feed on the surface was eliminated. The remaining silage was thoroughly homogenized. Next, 20 g samples were collected from five different sites of the silage bag for each. Each silage sample was mixed with 180 mL of pure water thoroughly and placed in a juicer for 1 min to crush the mixture. The alfalfa silage was filtered using four layers of qualitative filter paper and placed in a refrigerator at 4 °C for 24 h. The nutritional and fermentation indicators of the filtered alfalfa extract were determined. The contents of lactic acid (LA), acetic acid (AA), propionic acid (PA), butyric acid (BA) and the ratio of ammonia nitrogen to total nitrogen (AN/TN) were determined in accordance with the method stipulated in the reference standard DB15/T1458-2018 [22]. The calculation formula for the total nitrogen content is as follows: total nitrogen content = crude protein ÷ 6.25. The V-score scoring system was employed to assess the fermentation quality of alfalfa silage [23]. Then 100 g of alfalfa silage was taken and dried at 65 °C for 48 h before the detection of nutritional quality was initiated. The determination of dry matter (DM) was conducted by means of drying technology and in accordance with the recommended national standard GB/T 6435-2014 [24]; the proportion of crude protein (CP) was examined in accordance with the recommended national standard GB/T 6432-2018 [25]; the proportion of crude ash (ASH) was detected by the method of incineration under high-temperature conditions to obtain dry ash [26]. The determination of the proportions of neutral detergent fiber (NDF) and acid detergent fiber (ADF) was accomplished by means of the Van Soest detergent fiber analysis approach [27]; the detection method of water-soluble carbohydrates (WSC) in silage samples was determined in reference to the optimized anthrone-sulfuric acid method proposed by Xu et al. [28]. The pH of the filtrate was measured with the LAQUAtwin-pH-22 handheld precision pH meter in accordance with relevant protocols [29]. DDM is the digestible dry matter content in dry matter. The chemical composition of the feed was analyzed by near-infrared spectroscopy and the DDM was predicted in combination with the digestibility model. DCP is the crude protein content in dry matter. The calculation of RFV is usually based on dry matter (DM), crude protein (CP), acid detergent fiber (ADF) and neutral detergent fiber (NDF) in the feed [30]. The specific calculation method is defined as follows:

RFV = (DDM × DCP)/(ADF + NDF)

### 2.3. Statistical Analysis of Data

The interaction effect variance was assessed by the general linear model in Excel 2010 and SPSS 22.0 [31]. The results were presented as “mean ± standard deviation”. Duncan’s multiple comparison method was employed for an in-depth analysis of the data to examine whether there were significant differences among the treatments. *p* < 0.05 indicated a significant difference [32].

## 3. Results

### 3.1. The Nutritional Qualities of Different Alfalfa Silage

It can be observed from Table 3 that the CP content in treatment B of alfalfa silage was significantly higher than that in treatment A, treatment C, treatment D, and the F treatment (*p* < 0.05); the RFV of treatment B was significantly higher than that of the F treatment and treatment D (*p* < 0.05) and the WSC in treatment B was higher than that in the other treatments, though not significantly (*p* > 0.05); the NDF content in treatment B was significantly lower than that in the F treatment and treatment D (*p* < 0.05); compared with the other treatments, the NDF and ADF contents in treatment B decreased (*p* > 0.05).

It can be seen from Table 4 that the pH of the silage under the microbial inoculant in treatment B was significantly lower than that in the other treatments (*p* < 0.05). The AN/TN content in treatment A and the F treatment was significantly higher (*p* < 0.05) than that in B treatment. The LA content in treatment B was significantly higher than that in the other treatments (*p* < 0.05).

### 3.2. Comprehensive Analysis on the Quality of Alfalfa Silage

The quality of alfalfa silage was estimated with the aid of membership functions. The arithmetic average was taken to assess the silage quality of alfalfa and a comprehensive ranking was conducted based on the magnitude of the arithmetic average. The greater the arithmetic average, the higher the ranking and the better the silage quality. Carbohydrates (WSCs), lactic acid (LA) content, dry matter (DM) and crude protein (CP) were positively correlated with the silage quality. The calculation formula for the membership function value is as follows:

$$U(X_{j})=\frac{X_{j}-X_{min}}{X_{max}-X_{min}}$$

The pH, ammonia nitrogen/total nitrogen (AN/TN), acetic acid (AA) content, neutral detergent fiber (NDF), acid detergent fiber (ADF) and crude ash (ASH) are negatively correlated with the silage value of alfalfa. The calculation formula for the membership function value is as follows:

$$U(X_{j})=1-\frac{X_{j}-X_{min}}{X_{max}-X_{min}}$$

In the equation, U($X_{j}$) denotes the membership function value of a certain measured indicator; $X_{j}$ represents the measured value of this indicator; $X_{max}$ represents the maximum value of this indicator; and $X_{min}$ represents the minimum value of this indicator.

The silage membership function is actually a mathematical tool used to evaluate the quality of silage. Based on the principle of fuzzy mathematics, multiple quality indexes (such as pH, lactic acid content, ammonia nitrogen/total nitrogen ratio, etc.) of silage were evaluated comprehensively and a membership value was obtained. The membership value can directly reflect the quality of silage. Specifically, the silage membership function converts the actual measured value of each quality indicator into a membership between 0 and 1 by defining a membership function. These membership values are then average-weighted or other mathematical processing used and finally a comprehensive membership value is obtained. The closer this value is to 1, the better the quality of the silage [33]. It is a simple, rapid and relatively accurate comprehensive assessment method that has been widely applied in the screening of forage varieties and quality evaluation. The greater the average membership function value of silage is, the better the comprehensive performance of silage fermentation is. The membership function analysis of 11 core silage fermentation indicators of alfalfa is presented in Table 5. Evidently, the membership function value of silage fermentation under the treatment of treatment B microbial inoculant is the greatest, with an average membership function value of 0.84. Subsequently, it is treatment E, with an average membership function value of 0.51. The third is treatment D, with an average membership function value of 0.38. The silage effect of treatment A microbial inoculant is the poorest, with an average membership function value of 0.31. In this experiment, in light of the results of the membership function analysis, it can be observed that the silage quality of treatment B is the finest, with an average membership function value of 0.84.

## 4. Discussion

### 4.1. The Study on the Impact of Microbial Inoculants on the Nutritional Components of Silage Alfalfa

In view of the high buffering capacity of alfalfa, ensiling it alone is extremely difficult and yields poor results. Hence, adding an appropriate quantity of microbial inoculants to the sole ensiling raw materials is conducive to enhancing the overall quality of the silage [34]. CP is another crucial indicator for assessing the nutritional value of alfalfa silage and exerts a decisive influence on the growth, development and production performance of animals. During the ensiling process of alfalfa, the variation of CP content is affected by various factors and the reasonable application of silage microbial inoculants can effectively modulate this change [35]. The research conducted by Sun et al. [36] disclosed that lactic acid bacteria preparations, cellulase and lactic acid bacteria preparations plus cellulase all led to a significant decrease in the pH of silage and a remarkable increase in lactic acid content (*p* < 0.05). The addition of formic acid significantly reduced the pH, lactic acid and ammonia nitrogen content of silage (*p* < 0.05). The addition of lactic acid bacteria preparations or formic acid significantly enhanced the in vitro digestibility of dry matter (*p* < 0.05), and the mixed addition of lactic acid bacteria preparations and cellulase significantly elevated the in vitro digestibility of dry matter and crude protein (*p* < 0.05). In this experiment, the CP content of the alfalfa raw material was 25.51%. After the treatment with the addition of lactic acid bacteria, the CP content in treatment B significantly increased compared to the F treatment, which was in line with the findings of previous studies. This suggests that the silage inoculant of treatment B can significantly reduce the ammoniated crude protein in alfalfa silage and enhance the in vitro digestion rate of crude protein.

Fiber content holds an important position in alfalfa silage. The fiber content of silage directly influences the digestion and absorption capacity of crude protein [37]. The lower its content, the more readily CP can be digested, absorbed and utilized by ruminants [38]. The content of NDF can visually reflect the quality of cellulose. This research indicates that the NDF content of the test treatment with the addition of treatment B strains decreased significantly compared to the F treatment, suggesting that an appropriate increase in the addition quantity of treatment B lactic acid bacteria has a favorable effect on reducing the NDF content of alfalfa silage. Similar to previous studies [39], the addition of *Lactobacillus* can improve the nutritional value of alfalfa silage to a certain extent by protecting true protein and increasing the contents of rapidly degrading carbohydrate components and non-structural carbohydrates, and the addition of *Lactobacillus plantarum* and *Lactobacillus casei* has a better effect. Wang et al. [40] proved that silage treated with *Lactobacillus plantarum* could significantly reduce the contents of acid wash fiber, lignin, starch, ASH, acid wash insoluble protein and neutral wash insoluble protein, as well as pH value, volatile fatty acids, acetic acid, butyric acid and ammonia nitrogen. The silage effect of the *Lactobacillus plantarum* ACCC11016 treatment group was the best. It is not inconsistent with the results of this study that B additive contains LP and has the best silage effect. The results of this experiment are basically consistent with the research results of Liu et al. [41], which show that the simultaneous addition of *Lactobacillus buchneri* and cellulase can improve the fermentation quality and aerobic stability of silage corn stalks, indicating that adding lactic acid bacteria or *Lactobacillus buchneri* during silage can achieve ideal silage effects.

### 4.2. The Impact of Silage Inoculants on the Fermentation Characteristics of Alfalfa Silage

Upon opening the silage tank for fermentation, the environment transforms from anaerobic to aerobic, resulting in an increase in the number and activity of aerobic microorganisms. Yeast, molds and others utilize the lactic acid and residual nutrients generated by the silage to continuously generate heat, deteriorating the silage. Lactic acid bacteria are the most critical microbial species in this process. At this stage, adding microbial inoculants such as *Lactobacillus plantarum*, *Lactobacillus pentosus*, *Pediococcus acidilactici* and *Lactobacillus fermentum* can enable the silage to produce acetic acid, inhibit secondary fermentation and enhance the aerobic stability of the silage [42]. The aerobic stability of fermented feed refers to the duration when the core temperature of silage, upon exposure to air after opening the silo, rises by 2 °C above the external temperature. The pH is one of the significant indicators for assessing aerobic stability [43]. The types and activities of microorganisms growing and reproducing are the key influential factors for aerobic stability. Compared to treatment B, the elevated pH values in treatment A, treatment C and the F treatment might suggest that the aerobic microorganisms (such as yeasts and molds) in this treatment have stronger activities, resulting in the deterioration of the quality of the silage. During the ensiling process of *Medicago sativa* L., the variation of pH value plays a decisive role in the quality of silage. When the pH value is within an appropriate range, it can effectively restrain the growth of harmful microorganisms, thereby guaranteeing the quality of silage. Some scholars [44] analyzed the main reasons of secondary fermentation, including the cut of green stock being too long and low silage density; little silage is taken out every day and the thickness of the cut layer is very thin; silage is too dry, or harvest is too late and water content is too low; frost damage during harvest. On the first day after opening the cellar, the temperature rises to about 50 °C and the pH rises to 8 or higher at the same time, then the buffer capacity of silage is limited, silage passes through the neutral to slightly alkaline stage and finally completely decays. After the first temperature rise, the silage temperature is close to the air temperature, but the pH continues to rise, which is the result of the accumulation of volatile salt ground state nitrogen, which will further induce the proliferation of aerobic microorganisms. Further decomposition of proteins and amino acids. The research conducted by Yu Haoran et al. [45] has also verified that a low pH can restrain the activities of harmful bacteria and reduce the generation of ammonia nitrogen. During the entire fermentation process, the more rapidly lactic acid (LA) increases, the more rapidly the pH decreases. When the silage is of poor quality and the pH increases, it will facilitate the degradation of protein into amino acids, which are further decomposed into ammonia, resulting in an increase in the proportion of ammonia nitrogen (AN) in the silage. Adding *Lactobacillus plantarum* at this stage can effectively lower the content of AN. In this experiment, the LA content of treatment B was significantly higher than that of the other treatments. The pH of treatment B was significantly lower than that of the F treatment, treatment A, treatment C and treatment E, and the AN/TN was significantly lower than that of treatment A and the F treatment. These findings are in line with the results of previous studies, suggesting that the silage microbial inoculant of treatment B can notably reduce the pH and the rate of protein degradation of alfalfa silage and it is the optimal microbial inoculant for silage fermentation.

Although aerobic fermentation after the opening of the silage pit reduces the nutritional value of the feed, treatment B still managed to maintain a low pH. On the one hand, it might be attributed to the strong acid-producing capacity and high activity of *Lactobacillus plantarum* in the silage microbial inoculant of treatment B, which produced a large quantity of lactic acid to inhibit the growth of other pathogens. On the other hand, the facultative heterofermentative *Lactobacillus casei* (≥1 × 10^11^ CFU·g^−1^) in the silage microbial inoculant of treatment B increased the concentration of lactic acid bacteria. After the opening of the silage pit, it could convert LA to AA, achieving a further reduction in the pH of the silage. Compared with the F treatment, the overall quality of the silage in treatment B was maintained at a relatively superior level. Hao et al. [46] explored the influences of various silage microbial inoculants on the nutritional value and fermentation quality of alfalfa silage and discovered that the lactic acid bacteria treatment led to the lowest pH, the highest LA content and the optimal sensory evaluation of the silage [47]. In this experiment, the microbial inoculant of treatment B significantly enhanced the contents of CP and LA in alfalfa compared with other treatments, which is in accordance with the previous research findings. It is hypothesized that this outcome is associated with the high content and high activity of *Lactobacillus plantarum* and *Lactobacillus casei*, the main components in Xinlaiwang I—alfalfa silage inoculant, which can significantly improve the quality of the feed [48].

### 4.3. Comprehensive Analysis of the Silage Quality Based on the Membership Function

From the analysis of the membership function, it is observable that the average membership function value of silage is as follows: treatment B > treatment E > treatment D > treatment C > treatment F > treatment A. The addition of Xinlaiwang I—straw silage inoculant, Zhuang Le Mei silage inoculant and Baoshiqing did not manifest a significant improvement in the quality of alfalfa silage. The addition of KOFASIL S lactic acid bacteria silage inoculant (*Lactobacillus plantarum*) and the active ingredient (*Lactobacillus plantarum* and *Lactobacillus casei*) in Xinlaiwang I—alfalfa silage inoculant both enhanced the quality of alfalfa silage, yet the improvement effect of Xinlaiwang I—alfalfa silage inoculant on silage was more pronounced. Xinlaiwang I—alfalfa silage inoculant reduced the NDF and ADF contents of alfalfa to a certain extent and significantly increased the CP and LA contents (*p* < 0.05) and the RFV exhibited a significant difference from that of treatment A and treatment D (*p* < 0.05). RFV is approximately calculated based on the NDF and ADF contents in roughage. The lower the NDF and ADF contents, the better the quality. Consequently, the high RFV of silage is directly correlated with the low NDF content [49]. It can thus be inferred that Xinlaiwang I—alfalfa silage inoculant yields the optimal effect on alfalfa silage. It can effectively enhance the fermentation quality of alfalfa silage, conspicuously reduce AN/TN and inhibit the degradation of protein into non-protein nitrogen (non-protein nitrogen, NPN), ammonia nitrogen as well as lower the activity of protein-degrading enzymes. The reason for this result may be compared with that of Xinlaiwang I—straw silage inoculant, Zhuang Le Mei silage inoculant and Baoshiqing. Xinlaiwang I—alfalfa silage inoculant contains a greater quantity of *Lactobacillus plantarum* (≥1 × 10^11^) and in comparison with KOFASIL S lactic acid bacteria silage inoculant, it contains more *Lactobacillus casei* (≥1 × 10^11^), enabling it to significantly increase the LA content during silage fermentation, decrease the pH of the feed, minimize feed spoilage and nutrient loss, reduce the content of cellulose and anti-nutritional factors in the feed, enhance the storage stability of silage and improve the utilization rate of protein and heat energy of silage by livestock [50].

## 5. Conclusions

The results of the comprehensive analysis of the membership functions indicate that, in contrast to other silage microbial inoculants, Xinlaiwang I—alfalfa silage inoculant can prominently enhance the LA and CP contents of alfalfa silage, significantly lower the pH of the silage and enhance the nutritional value and fermentation quality of the silage. Xinlaiwang I—alfalfa silage inoculant can significantly ameliorate the fermentation quality, aerobic stability and nutritional value of alfalfa silage and is more appropriate to be used as an alfalfa silage microbial inoculant.

## Figures and Tables

**Table 1:** The nutrient composition of alfalfa.

DM (FM%)	CP (DM%)	NDF (DM%)	ADF (DM%)	WSC (DM%)	ASH (DM%)	RFV (%)
38.37	25.51	33.67	25.41	5.77	12.06	191.33

Note: dry matter, DM; fresh alfalfa material, FM; crude protein, CP; neutral detergent fiber, NDF; acid detergent fiber, ADF; water soluble carbohydrate, WSC; crude ash, ASH; Relative Feeding Value, RFV.

**Table 2:** Source, composition and dosage of silage inoculant.

Microbial Colony/(CFU·g^−1^) and Source	Xinlaiwang I—Straw Silage Inoculant	Xinlaiwang I—Alfalfa Silage	Zhuang Le Mei Silage Inoculant	Baoshiqing	KOFASIL S Lactic Acid Bacteria Silage Inoculant
*Lactobacillus plantarum*	≥1 × 10^10^	≥1 × 10^11^	≥1.6 × 10⁹	≥2.5 × 10^10^	≥1 × 10^11^
*Pediococcus pentosaceus*	≥1 × 10^10^	ND	ND	≥2.5 × 10^10^	ND
*Lactobacillus buchneri*	≥1 × 10^10^	ND	≥4.0 × 10⁸	≥2.5 × 10^10^	ND
*Lactobacillus casei*	ND	≥1 × 10^11^	ND	ND	ND
Water content	ND	ND	<10.0%	ND	ND
Source	China Nanjing Xinlaiwang Biotech Co., Ltd. (Nanjing, China)	China Nanjing Xinlaiwang Biotech Co., Ltd. (Nanjing, China)	China Sichuan Gaofuji Bio-Technology Co., Ltd. (Chengdu, China)	China Newman Agricultural Trading Shanghai Co., Ltd. (Shanghai, China)	China Edcon Dalian Environmental Protection Products Co., Ltd. (Dalian, China)
Dosage	2 g/ton	2 g/ton	5 g/ton	1 g/ton	1 g/ton

Note: ND means not detected.

**Table 3:** Effects of different microbial inoculants on the nutritional quality of alfalfa (% DM).

Item	F	A	B	C	D	E
DM (% FM)	38.80 a ± 0.11	37.13 c ± 0.21	37.54 bc ± 1.11	38.08 ab ± 0.06	37.30 bc ± 0.07	38.18 ab ± 0.08
CP (% DM)	23.30 b ± 0.56	23.40 b ± 0.72	25.20 a ± 0.75	23.63 b ± 0.38	23.60 b ± 0.35	24.20 ab ± 0.72
NDF (% DM)	40.64 a ± 1.55	40.24 ab ± 2.24	37.57 b ± 1.04	39.78 ab ± 0.90	41.85 a ± 0.85	40.17 ab ± 1.56
ADF (% DM)	29.32 a ± 1.02	28.80 a ± 2.44	27.29 a ± 0.88	28.73 a ± 0.70	29.49 a ± 0.59	29.21 a ± 1.44
WSC (% DM)	3.36 a ± 0.29	3.68 a ± 0.77	3.72 a ± 0.55	3.37 a ± 0.54	3.51 a ± 0.32	2.92 a ± 0.38
ASH (% DM)	11.53 a ± 1.00	10.75 a ± 1.00	11.53 a ± 1.00	12.04 a ± 1.00	11.73 a ± 0.64	10.66 a ± 0.90
RFV (%)	145.79 b ± 7.27	148.51 ab ± 13.16	162.41 a ± 5.90	149.92 ab ± 4.69	141.19 b ± 2.13	148.08 ab ± 8.51

Note: dry matter, DM; fresh alfalfa material, FM; crude protein, CP; neutral detergent fiber, NDF; acid detergent fiber, ADF; water soluble carbohydrate, WSC; crude ash, ASH; Relative Feeding Value, RFV. The difference was significant if the shoulder letters of the same column were different (*p* < 0.05), but the difference was not significant (*p* > 0.05).

**Table 4:** Effects of different lactic acid bacteria treatment on the quality of alfalfa silage.

Item	F	A	B	C	D	E
pH	4.91 a ± 0.04	4.94 a ± 0.08	4.65 c ± 0.02	4.96 a ± 0.06	4.70 bc ± 0.03	4.75 b ± 0.01
AN/TN (%)	4.70 a ± 5.49	4.54 a ± 4.46	2.66 b ± 5.54	4.74 a ± 2.01	4.49 a ± 6.32	4.29 a ± 3.00
LA (%FM)	2.15 b ± 15.01	2.00 b ± 8.53	5.57 a ± 9.83	1.83 b ± 3.04	2.32 b ± 10.78	1.89 b ± 12.83
AA (%FM)	1.43 b ± 37.94	1.12 b ± 8.74	2.83 a ± 32.61	1.01 b ± 11.42	1.21 b ± 17.69	1.05 b ± 25.86
PA (%FM)	ND	ND	ND	ND	ND	ND
BA (%FM)	ND	ND	ND	ND	ND	ND

Note: ammonia nitrogen/total nitrogen, AN/TN; lactic acid, LA; acetic acid, AA; propionic acid, PA; butyric acid, BA. The acronym used in the full article is the same as the one used in these notes. The difference was significant if the shoulder letters of the same column were different (*p* < 0.05), but the difference was not significant (*p* > 0.05).

**Table 5:** Nutrition content composition of silage feeds of alfalfa silage in different LAB treatments.

Item	F	A	B	C	D	E
DM	1	0	0.25	0.57	0.1	0.63
CP	0	0.05	1	0.17	0.16	0.47
WSC	0.55	0.95	1	0.56	0.74	0
LA	0	0.40	1	0.13	0.41	0.13
RFV	0.22	0.34	1	0.41	0	0.32
NDF	0.28	0.38	1	0.48	0	0.39
ADF	0.08	0.31	1	0.35	0	0.13
pH	0.16	0.06	1	0	0.84	0.68
AN	0.28	0	1	0.49	0.87	0.89
ASH	0.37	0.93	0.37	0	0.22	1
AA	0.60	0	0.64	0.42	0.85	1
Mean	0.32	0.31	0.84	0.33	0.38	0.51
Ranking	5	6	1	4	3	2

## Data Availability

The data presented in this study are available on request from the corresponding authors due to privacy or ethical restrictions.

## Abbreviations

The following abbreviations are used in this manuscript:

FM	Fresh matter
DM	Dry matter
CP	Crude protein
RFV	Relative Feeding Value
NDF	Neutral detergent fiber
ADF	Acid detergent fiber
WSC	Water soluble carbohydrate
ASH	Crude ash
LA	Lactic acid
AA	Acetic acid
PA	Propionic acid
BA	Butyric acid
